# Safety and efficacy of a probiotic-containing infant formula supplemented with 2’-fucosyllactose: a double-blind randomized controlled trial

**DOI:** 10.1186/s12937-022-00764-2

**Published:** 2022-02-22

**Authors:** Philippe Alliet, Yvan Vandenplas, Paola Roggero, Sabine N. J. Jespers, Stefaan Peeters, Jean-Philippe Stalens, Guus A. M. Kortman, Mailis Amico, Bernard Berger, Norbert Sprenger, Colin I. Cercamondi, Giovanni Corsello

**Affiliations:** 1 Department of Pediatrics, Jessa Hospital, Hasselt, Belgium; 2 KidZ Health Castle, Vrije Universiteit Brussel, UZ Brussel, Brussels, Belgium; 3 Department of Clinical Sciences and Community Health, University of Milan, Milan, Italy; 4 Clinique Et Maternité Sainte-Elisabeth, Namur, Belgium; 5 Algemeen Stedelijk Ziekenhuis Aalst, Aalst, Belgium; 6 Department of Pediatrics, Centre Hospitalier de Wallonie Picarde, Tournai, Belgium; 7 NIZO Food Research BV, Ede, The Netherlands; 8 Biostatistics & Data, Nestlé Research, Société des Produits Nestlé S.A., Lausanne, Switzerland; 9 Nestlé Institute of Health Sciences, Nestlé Research, Société des Produits Nestlé S.A., Lausanne, Switzerland; 10 Nestlé Product Technology Center – Nutrition, Société des Produits Nestlé S.A., Vevey, Switzerland; 11 Department of Health Promotion Sciences Maternal and Infant Care, University of Palermo, Palermo, Italy

**Keywords:** Infant formula, Human milk oligosaccharides, 2’fucosyllactose, Growth, Gut microbiome

## Abstract

**Background:**

Human milk oligosaccharides (HMOs) have important and diverse biological functions in early life. This study tested the safety and efficacy of a starter infant formula containing *Limosilactobacillus (L.) reuteri* DSM 17938 and supplemented with 2’-fucosyllactose (2’FL).

**Methods:**

Healthy infants < 14 days old (*n* = 289) were randomly assigned to a bovine milk-based formula containing *L. reuteri* DSM 17938 at 1 × 10^7^ CFU/g (control group; CG) or the same formula with added 1.0 g/L 2’FL (experimental group; EG) until 6 months of age. A non-randomized breastfed group served as reference (BF; *n* = 60). The primary endpoint was weight gain through 4 months of age in the formula-fed infants. Secondary endpoints included additional anthropometric measures, gastrointestinal tolerance, stooling characteristics, adverse events (AEs), fecal microbiota and metabolism, and gut immunity and health biomarkers in all feeding groups.

**Results:**

Weight gain in EG was non-inferior to CG as shown by a mean difference [95% CI] of 0.26 [-1.26, 1.79] g/day with the lower bound of the 95% CI above the non-inferiority margin (-3 g/day). Anthropometric Z-scores, parent-reported stooling characteristics, gastrointestinal symptoms and associated behaviors, and AEs were comparable between formula groups. Redundancy analysis indicated that the microbiota composition in EG was different from CG at age 2 (*p* = 0.050) and 3 months (*p* = 0.052), approaching BF. Similarly, between sample phylogenetic distance (weighted UniFrac) for BF vs EG was smaller than for BF vs CG at 3-month age (*p* = 0.045). At age 1 month, *Clostridioides difficile* counts were significantly lower in EG than CG. *Bifidobacterium* relative abundance in EG tracked towards that in BF. Fecal biomarkers and metabolic profile were comparable between CG and EG.

**Conclusion:**

*L. reuteri*-containing infant formula with 2’FL supports age-appropriate growth, is well-tolerated and may play a role in shifting the gut microbial pattern towards that of breastfed infants.

**Trial Registration:**

The trial was registered on ClinicalTrials.gov (NCT03090360) on 24/03/2017.

**Supplementary Information:**

The online version contains supplementary material available at 10.1186/s12937-022-00764-2.

## Background

The third most abundant solid component of breast milk after lactose and lipids is structurally diverse oligosaccharides, known collectively as human milk oligosaccharides (HMOs). Mature breast milk contains approximately 5–20 g/L HMOs of > 160 different known structures [1, 2]. HMOs play diverse and important roles in the development of infants. They have been shown to support the establishment and maintenance of a balanced gut microbiota, a key factor in the development of the mucosal immune system [2]. HMOs may also play an important role in immune protection by providing anti-adhesive antimicrobial effects, regulating intestinal epithelial cell response, and modulating immune response via direct effects on immune cell populations and cytokine secretion [2–4]. Additionally, preclinical and observational data suggests potential beneficial effects of HMOs on brain development [5, 6].

Types and concentrations of HMOs vary considerably according to women’s genetic profile and time of lactation [7]. One abundant HMO commonly found in breast milk is 2’-fucosyllactose (2’FL). In secretor positive mothers, concentration of 2’FL in mature breast milk ranges from 0.9 – 4.0 g/L with an average of approximately 2.0 g/L [7–11]. In observational studies, 2’FL has been associated with reduced risk of illness or lower mortality in breastfed infants [12–16]. In randomized controlled trials (RCT), infant formulas containing 2’FL in combination with lacto-N-*neo*tetraose (LNnT) or galactooligosaccharides (GOS) have been shown to support age-appropriate growth [17, 18]. Infant formula with 2’FL (1 g/L) and LNnT (0.5 g/L) beneficially impacted gut microbiota development [19] and formulas containing either 0.2 g/L 2’FL plus 2.2 g/L GOS or 1.0 g/L 2’FL plus 1.4 g/L GOS may support development of the immune system [20]. These effects might translate into a reduced risk for certain infections in formula-fed infants and related to that to a reduced use of antibiotics and antipyretics as reported in the trial with 2’FL and LNnT supplemented infant formula [18]. Infant formulas with 2’FL (0.25 g/L) in combination with *Bifidobacterium (B.) lactis* Bb12 or *Limosilactobacillus (L.) reuteri* DSM 17938 (formerly *Lactobacillus reuteri* DSM 17938) showed good gastrointestinal (GI) tolerance or a potential soothing effect in infants with parent-perceived fussiness, respectively [21, 22]. A recent real-world evidence study reported good GI tolerance and age-appropriate growth in infants exclusively fed formula containing 2’FL (1 g/L), LNnT (0.5 g/L) and *L. reuteri* DSM 17938 or infants who received the formula and breast milk [23].

*L. reuteri* has been found in breast milk as well as in the human gut [24]. Colonization with *L. reuteri* in the GI tract has been associated with beneficial health effects [24]. A previous study reported high *L. reuteri* colonization rates in breastfed infants receiving *L. reuteri* DSM 17938 supplementation (10^8^ CFU/day) [25]. Meta-analyses on *L. reuteri* supplementation with drops, including DSM 17938 and ATCC 55730 strains, have shown a reduction of crying and/or fussing time in mainly breastfed infants [26, 27]. Infant formula supplemented with *L. reuteri* DSM 17938 has been shown to be safe [28] and to beneficially alter microbiota development of caesarian-delivered infants [29]. To date, RCT data for formula containing both *L. reuteri* and 2’FL is scarce. The aim of this study was therefore to assess growth, GI tolerance, as well as the gut microbiome and intestinal maturation of infants fed an experimental formula containing 2’FL at a level found in breast milk and *L. reuteri* DSM 17938 in comparison to infants fed a control formula containing only *L. reuteri*. A group of non-randomized breastfed infants served as reference, primarily for the gut microbiome and intestinal maturation outcomes.

## Methods

### Study aim, design, and setting

Safety and efficacy of infant formula with *L. reuteri* DSM 17938 and 2’FL was evaluated in a double-blind RCT of healthy term infants conducted between March 2017 and May 2019 in five centers in Belgium (KidZ Health Castle, Brussels; Clinique et Maternité Sainte-Elisabeth, Namur, Algemeen Stedelijk Ziekenhuis Aalst, Aalst; Centre Hospitalier de Wallonie Picarde, Tournai; Jessa Hospital, Hasselt) and two in Italy (University of Palermo, Palermo; University of Milan, Milan). Two randomized formula-fed groups and a non-randomized breastfed reference group (BF) were included. Formula-fed infants at ≤ 14 days of age were randomized to either the experimental group (EG) or control group (CG) in a 1:1 allocation ratio, stratified by center, sex, and mode of delivery (Cesarean- or vaginal-born). Randomization was completed using Medidata Balance with the dynamic allocation algorithm. Investigators, study staff, and parents/caregivers (hereafter, “parents”) were blinded to the study formulas. Formulas were coded by the manufacturer (Nestle Product Technology Center, Konolfingen, Switzerland) using three meaningless codes within each formula group (i.e., a total of 6 meaningless codes).

CG received a standard bovine milk-based whey predominant formula with an energy density of 670 kcal/L containing 75 g/L lactose, 34 g/L fat, 14 g/L protein (60:40 whey:casein ratio), and *L. reuteri* DSM 17938 at 1 × 10^7^ CFU/g (translating to approximately 1 × 10^9^ CFU/day in 4 months old infants). EG received the same formula supplemented with 1 g/L 2’FL which is within the range of 2'FL concentrations generally observed in breastmilk [7–11]. The intervention period was approximately 180 days. Formula-fed infants were required to exclusively consume the study formula until at least 4 months of age, after which progressive introduction of complementary foods or liquids was allowed. Mothers of infants enrolled in BF were asked to continue exclusive breastfeeding up to at least 4 months.

Infants completed study visits at baseline (≤ 14 days of age) and then at 1, 2, 3, 4, and 6 months of age. At baseline, demographic characteristics were collected and parents completed a 1-day GI symptom diary to retrospectively document stool characteristics, GI tolerance and associated behaviors, and formula intake for the day before the baseline visit. At each visit, anthropometrics (weight, length, and head circumference) were collected. Parents completed a 3-day GI symptom diary at home to prospectively document stool characteristics, GI tolerance and associated behaviors as well as feeding information for the 3 consecutive days prior to each post-baseline visits. Fecal samples were collected at baseline (before the intervention started) and at 1, 2, and 3 months of age. Parent-reported and physician-confirmed adverse events (AEs) were recorded throughout the study.

### Participant characteristics

Healthy, term (37–42 weeks gestation) infants aged ≤ 14 days at enrollment with a birth weight between 2500 and 4500 g whose parents signed the informed consent form were included in the study. At the time of enrollment, infants in the formula groups and BF were required to be exclusively formula-fed or breastfed, respectively. Infants who received complementary foods or liquids, participated in other clinical trials, had a medical condition that could increase risks associated with study participation or interfere with interpretation of results (such as major congenital malformations, congenital infections, history of neonatal intensive care unit admission, or other severe medical or laboratory abnormality), and infants who received medications prior to enrollment that may affect study results (growth, fat digestion, absorption, and/or metabolism; stool characteristics and gastric acid secretion) were excluded.

### Outcome measures

The primary outcome was weight gain between baseline and age 4 months in the formula-fed infants as recommended in guidelines from the American Academy of Pediatrics Task Force on Clinical Testing of Infant Formulas [30]. Weight gain (g/day) was calculated as the difference in infant weight between the baseline and 4 months visits divided by the number of days between the two visits. Secondary outcomes included anthropometric z-scores, stool characteristics, GI tolerance and associated behaviors, fecal microbiota, fecal metabolism, fecal markers of gut immunity and gut health, and parent-reported and physician-confirmed AEs.

Infant weight was measured without clothing or diaper on a calibrated electronic scale to the nearest 10 g. Recumbent length was recorded using a calibrated length board to the nearest 0.1 cm, and head circumference using a standard non-elastic measuring tape to the nearest 0.1 cm. Corresponding z-scores for weight-for-age, length-for-age, weight-for-length, and head circumference-for-age were calculated using the World Health Organization (WHO) Child Growth Standards [31]. Stool characteristics and GI tolerance and associated behaviors were captured in the study diaries. Stool frequency was assessed as the number of stools per day, difficulty in passing stool as number of stools difficult to pass per day, and stool consistency was recorded using a validated 5-point scale (1 = watery, 2 = runny, 3 = mushy soft, 4 = formed, 5 = hard) provided in a pictorial representation to parents [32]. The frequency of spitting-up/vomiting and flatulence episodes per day were recorded on a categorical scale (1 time; 2–3 times; 4–6 times; > 7 times). Categorical scales were also used to assess the daily durations of crying or fussing (< 10 min; 10–30 min; > 30 min to 1 h; > 1–2 h; > 2–3 h > 3 h), sleeping (0–8 8–12, 12–16, 16–20, 20–24 h) or severity of spitting-up/vomiting (1 teaspoon or less; 1 tablespoon; 2 tablespoons; about half of the feeding; more than half of the feeding). AEs were recorded during clinic visits and phone calls in between the visits throughout the study, and 14 days after completion of the study feeding. All parent-reported and physician-confirmed AEs were categorized using the Medical Dictionary for Regulatory Activities (MedDRA) preferred terms.

### Fecal DNA extraction and 16S rRNA gene sequencing

DNA isolation, including vigorous bead-beating steps, was performed as described previously [33]. Barcoded amplicons from the V3–V4 region of 16S rRNA genes were generated using a 2-step polymerase chain reaction (PCR) and according to previously described methods [33]. For the library PCR step in combination with sample-specific barcoded primers, purified PCR products were shipped to BaseClear BV (Leiden, The Netherlands). PCR products were checked on a Bioanalyzer (Agilent) and quantified. This was followed by multiplexing, clustering and sequencing on an Illumina MiSeq with the paired-end (2x) 300 bp protocol and indexing. The sequencing run was analyzed with the Illumina CASAVA pipeline (v1.8.3) with de-multiplexing based on sample-specific barcodes. Sequence reads of too low quality (only “passing filter” reads were selected) and reads containing adaptor sequences or PhiX control were discarded from the raw sequencing data. On the remaining reads, a quality assessment was performed using FastQC version 0.10.0. (http://www.bioinformatics.babraham.ac.uk/projects/fastqc/).

Sequences of the 16S rRNA gene were analyzed using a workflow based on Qiime 1.8 [34]. On average, 29,570 (range 3,308 – 148,882) 16S rRNA gene sequences per sample were analyzed. We performed operational taxonomic unit (OTU) clustering (open reference), taxonomic assignment and reference alignment with the pick_open_reference_otus.py workflow script of Qiime, using uclust as clustering method (97% identity) and GreenGenes v13.8 as reference database for taxonomic assignment. Reference-based chimera removal was done with Uchime [35]. The RDP classifier version 2.2 was performed for taxonomic classification [36].

### Pathogenic bacteria species by quantitative PCR (qPCR)

Detection and quantification of selected genes of opportunistic pathogenic bacteria species was done with isolated DNA from fecal samples using validated commercial Genesig® qPCR kits from Primerdesign Ltd™ (*Klebsiella (K.). pneumonia*, *Salmonella* species*, Campylobacter (C.) jejuni,* and *C. coli*) or based on previously described methods (*Clostridioides (C.) difficile* [37], *Clostridium (C.) perfringens* [38], Enteropathogenic *Escherichia coli* (EPEC), Enterotoxigenic *Escherichia coli* (ETEC) heat-labile toxin and ETEC heat-stable toxin [39]).

### Fecal pH, organic acid and biomarker analysis

Fecal pH was assessed using an electrode-fitted pH meter after suspending 0.5 g (fresh weight) of fecal sample in 2 mL milliQ water. Organic acids (lactate, acetate, propionate, butyrate, isobutyrate, valerate, isovalerate) were determined by high performance anion‐exchange chromatography with UV and refractive index detection according to a modified and previously described method [40].

Commercially available ELISA kits were used to analyze fecal markers of intestinal immunity and health including secretory immunoglobulin A (sIgA), myeloperoxidase, calprotectin, human beta defensin (all Immundiagnostik AG, Bensheim, Germany) and neopterin (IBL, Hamburg, Germany).

### Statistics

Sample size was calculated considering the primary (weight gain) and key secondary endpoints (*Bifidobacterium* and *Peptostreptococcaceae* abundance) using a hierarchical approach to control for multiplicity. A non-inferiority margin of -3 g/day was used to demonstrate non-inferiority in weight gain according to guidelines from the American Academy of Pediatrics [30] and a SD of 6.0 was assumed [18, 41]. Based on a previous study [19], superior *Bifidobacterium* abundance in EG vs. CG was assumed at age 3 months (difference of 0.48 in the logit of the proportion of *Bifidobacterium* with a SD of 1.06). Similarly, inferior abundance in EG vs. CG was assumed at age 3 months for *Peptostreptococcaceae*, a family to which opportunistic pathogens, such as *C. difficile* belong [42] (difference of -0.55 in the logit of the proportion of *Peptostreptococcaceae* with a SD of 1.15). The smallest effect to be demonstrated was inferiority of *Peptostreptococcaceae*, requiring a sample size of 210 formula-fed infants (105/formula group) to reach a power of 80% at α = 0.05. An a priori power calculation indicated 95% power to detect non-inferior weight gain and 86% power to detect superiority of *Bifidobacterium* with 105 completed infants per formula-fed group. Assuming 35% loss to follow-up, approximately 280 formula-fed infants were enrolled. Sample size of BF (*n* = 60) was not based on statistical consideration but instead determined by practical and logistical feasibility.

The primary endpoint of weight gain between baseline and 4 months of age was analyzed using analysis of covariance (ANCOVA) adjusted for baseline weight, sex, mode of delivery, and study center. Non-inferiority was determined if the lower bound of the 95% CIs for the intervention difference was above -3 g/day. The primary endpoint was analyzed in the full-analysis set (FAS) and per-protocol (PP) populations. The FAS population included all formula-fed infants randomly assigned to CG or EG who took at least one feeding of the assigned formula and who had weight measurements available at baseline and age 4 months. The PP population consisted of all infants included in the FAS that were compliant with the feeding regimen on ≥ 80% of the days from baseline until age 4 months. A compliant day was defined as a day on which only the study formula was exclusively fed (i.e. no other formulas, breastmilk, complementary foods or liquids, such as water or tea).

Secondary endpoints were analyzed in the intention-to-treat (ITT) population except for the gut microbiota or AEs. The ITT population was defined as all infants randomly assigned to EG or CG, or infants enrolled in BF, independently from the actual feeding. Gut microbiota data (16S rRNA) was analyzed in the infants who provided stool samples and were compliant with the study feeding regimen on ≥ 80% of the days until the study visit at age 3 months. AEs were analyzed in the safety analysis set which included all randomized formula-fed infants or enrolled BF infants with at least one documented feeding of the randomly assigned study formula or breastmilk, respectively. A robust ANCOVA adjusted for baseline value, mode of delivery, sex, study center, and visit was used to compare the changes from baseline in the anthropometric z-scores between the feeding regimens. A Mixed Model Repeated Measures (MMRM) adjusted for the same variables as the ANCOVA was used to compare stool consistency and frequency, spitting-up/vomiting and flatulence episodes, crying and sleep duration. Difficulty in passing stool (as number of infants having at least one stool difficult to pass over the 3-day collection period) and dichotomized severity of spitting-up/vomiting and duration of fussiness were analyzed using a logistic regression model adjusted for the aforementioned variables. A scoring approach was used for outcomes for which data was collected on a categorical scale and scores were compared between the feeding groups. Incidence of AEs and use of concomitant medications were compared between formula groups using Fisher’s exact test.

qPCR targets were analyzed using log-transformed data in a MMRM adjusted for baseline concentration, sex, mode of delivery, antibiotic use, study center, and visit. Fecal pH, acetate, butyrate, lactate, and propionate were analyzed using log-transformed data in a linear mixed model adjusted for the same variables as the qPCR targets. Due to the low number of infants with detectable concentration of valerate, isovalerate and isobutyrate, odds ratios of the presence of these fecal organic acids were calculated using a logistic regression adjusted for the aforementioned variables. Fecal biomarkers were evaluated using log-transformed data in a linear mixed model adjusted for baseline concentration, sex, mode of delivery, study center and visit. All aforementioned analyses were conducted using R version 3.2.3 (2015–12-10).

Statistical tests for the 16S rRNA gene sequences were performed as implemented in SciPy (http://www.scipy.org/), downstream of the Qiime-based workflow. We tested for between-group differences per time point in alpha phylogenetic diversity (Faith’s index, PD_whole tree metric) with the non-parametric Kruskal–Wallis test and Dunn’s posthoc test, as implemented in Graphpad Prism 5.01 (San Diego, CA, USA). Beta diversity (weighted UniFrac; for each infant in a group the average distance to all infants in another group was calculated) per time point was compared with the non-parametric Mann–Whitney U test (one-tailed), as implemented in Graphpad Prism 5.01 (San Diego, CA, USA). Between group-differences of pre-selected single taxa of importance in the studied age range, were assessed per time point using the non-parametric Kruskal–Wallis test with Dunn’s posthoc test.

We performed multivariate redundancy analyses (RDA) on the gut microbiota composition as assessed by 16S rRNA gene sequencing in Canoco version 5.12 using default settings of the analysis type “Constrained” [43]. Relative abundance values of OTUs were used as response data, and metadata as explanatory variable. For visualization purposes, families or genera, rather than OTUs, were plotted as supplementary variables. Variation explained by the explanatory variables corresponds to the classical coefficient of determination (R2) and was adjusted for degrees of freedom (for explanatory variables) and the number of cases. Canoco determines RDA significance by permutating (Monte Carlo) the sample status. Per time point and sample set, confounding factors were first identified by RDA. Statistically significant confounders were included as covariates in subsequent analyses. Hence, partial RDA was employed to correct for covariance where relevant, covariates were first fitted by regression and then “partialled out” (removed) from the ordination. All tests were performed using a significance level of 5% with a two-sided p-value (except for weighted UniFrac analysis for which a one-sided *p*-value was used).

## Results

### Study Subjects

**Fig. 1:**
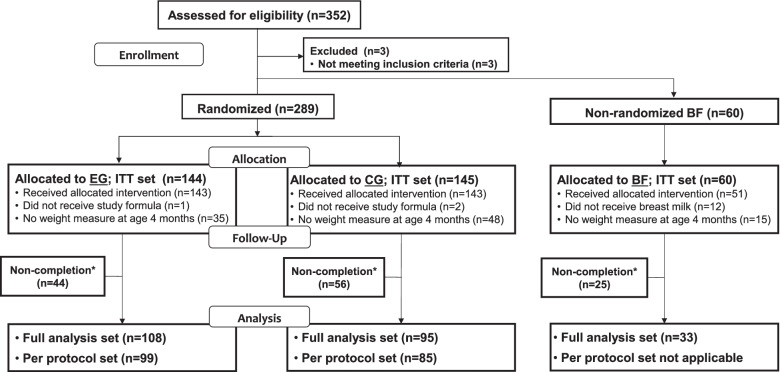
Flow chart of study subject disposition. BF, breastfed group; CG, control group; EG, experimental group; ITT, intention-to-treat. *Non-completion includes infants that dropped-out of the study before the 6 months of age visit. Infants dropping out between the 4 and 6 months of age visits might still be part of the full analysis or per protocol set. The numbers of the per protocol sets are for the primary endpoint which was not assessed in BF

A total of 289 formula-fed infants were randomized to EG (*n* = 144) and CG (*n* = 145) and 60 infants were enrolled into BF (Fig. 1). The FAS, including all randomized infants who took at least one feeding of the study formula with weight measurements available at baseline and 4 months, was comprised of 108 infants in EG, 95 in CG, and 33 in BF. The drop-out rate was 30% and 39% for EG and CG, respectively, and 41% for BF. Approximately half of the drop-outs (18% of the enrolled infants) were parents withdrawing consent without explanation.

Baseline characteristics were generally comparable among the three groups (Table 1). Infants were approximately 5–6 days of age at enrollment and slightly less than half were male. A higher proportion of delivery via Caesarian-section and lower proportion of mothers completing college was found among formula-fed infants compared to BF.

**Table 1:** Baseline characteristics of study participants in the three feeding groups in the intention-to-treat population

Characteristic	Experimental group (*n* = 144)	Control group (*n* = 145)	Breastfed group (*n* = 60)
Age at enrollment, days	6.8 (3.98)	6.7 (3.80)	4.8 (3.83)
Sex, % male	47.9%	46.9%	41.7%
Days breastfed since birth	3.3 (2.95)	4.4 (3.53)	4.8 (3.83)
Days fed formula since birth	6.3 (3.78)	6.3 (3.91)	N/A
Race, % Caucasian	97.9%	95.2%	90.0%
Gestational age, weeks	39.3 (1.17)	39.1 (1.03)	39.3 (1.02)
Delivery method, % Caesarean	33.3%	33.8%	20.0%
Birth weight, kg	3.3 (0.44)	3.4 (0.42)	3.4 (0.43)
Birth length, cm	49.8 (1.71)	50.0 (2.02)	50.2 (2.04)
Birth head circumference, cm	34.4 (1.42)	34.3 (1.37)	34.6 (1.28)
Mother’s age, years	31.0 (5.42)	30.9 (5.73)	30.9 (4.96)
Mother’s educational attainment, % completed college	20.8%	20.7%	40.0%

Data are presented as mean values (standard deviations) or percentages

### Growth

**Fig. 2:**
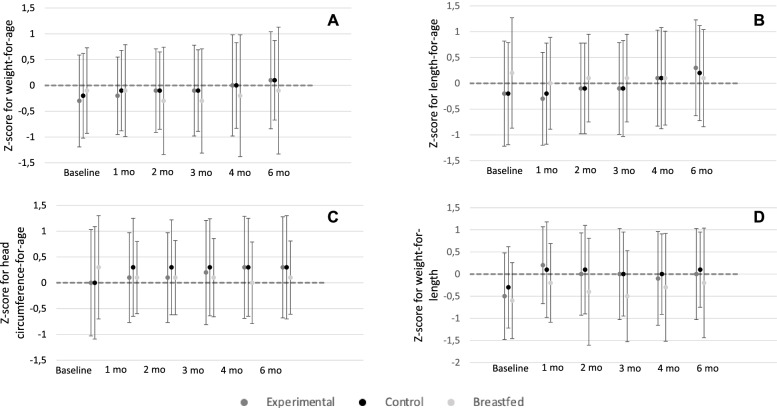
Anthropometric z-scores in the intention-to-treat population for **a** weight-for-age, **b** length-for-age, **c** head-circumference-for-age, and **d** weight-for-length from baseline (≤ 14 days of age) to 6 months of age. Values are means with SD as whiskers. No statistical differences between the feeding groups were observed at any time point using a robust ANCOVA comparing the changes from baseline in the anthropometric z-scores between the feeding groups and adjusted for baseline value, mode of delivery, sex, study center, and visit

In the FAS population, adjusted LS mean (SE) for weight gain between baseline and 4 months of age was 29.15 (0.65) g/day for EG and 28.89 (0.71) g/day for CG (Table 2). The LS mean difference (95% CI) in weight gain between the two groups was 0.26 (-1.26; 1.79) g/day (*p* = 0.736), with the lower limit of the 95% CI above the predefined non-inferiority margin of -3 g/day (*p* < 0.0001), indicating non-inferior weight gain in EG compared to CG. Results in the PP population (Table 2, LS mean difference 0.32 [95% CI: -1.33; 1.96]) also demonstrated non-inferior weight gain in EG comparted to CG. Anthropometric z-scores were comparable between the three feeding regimens. Overall, the z-scores for all groups tracked closely with the median of the WHO growth standards at all study visits (Fig. 2).

**Table 2:** Comparison of weight gain from baseline (≤ 14 days of age) to 4 months of age between formula-fed groups

**Population**	**Groups**	**Weight gain, g/day LS Mean (SE)**	**Difference between groups** **(Experimental-control)**^**a**^			***P*-value for non-inferiority**
			**Estimate**	**95% CI**	***P*-value**	
FAS	Experimental (*N* = 144)	29.15 (0.65)	0.26	-1.26; 1.79	0.736	< 0.001
	Control (*N* = 145)	28.89 (0.71)				
PP	Experimental (*N* = 99)	29.13 (0.70)	0.32	-1.33; 1.96	0.704	< 0.001
	Control (*N* = 85)	28.81 (0.79)				

(LS mean values and standard errors; 95% confidence interval)

*CI* Confidence interval, *FAS* Intention-to-treat, *PP* per protocol, *SE* Standard Error

^a^ From ANCOVA model with formula group, baseline weight, sex, mode of delivery, and study center as covariates

### Formula intake, GI tolerance and adverse events

At 4 months of age, mean (SD) daily formula intake was 790 (190) mL/day in EG and 780 (156) mL/day in CG, while mean (SD) daily number of feedings was 4.9 (1.1) and 4.9 (1.0), respectively. GI tolerance indicators and associated behaviors are presented in supplementary Tables 1 and 2 (Additional file 1). The overall stool consistency scores were similar between EG and CG (LS means [95% CI]: 2.7 [2.6; 2.8] vs. 2.8 [2.6; 2.9]; *p* = 0.278) with significantly higher scores than BF (1.9 [1.7; 2.1]; *p* < 0.0001). Overall stool frequency was comparable between EG and CG (LS means [95% CI]: 1.6 [1.5; 1.7] vs. 1.6 [1.5; 1.7]; *p* = 0.978). BF had significantly more stools (1.8 [1.7;2.1]; *p* < 0.05). No significant difference was observed between any of the groups for the number of infants with difficulty in passing stool during the period of exclusive formula or breast milk feeding. The overall frequency of spitting-up/vomiting per day or severity of spitting-up/vomiting was comparable between groups. Overall scores for frequency of flatulence were similar in the formula groups (LS means [95% CI]: 2.7 [2.5; 2.8] vs. 2.7 [2.6; 2.9]; *p* = 0.625), though significantly higher than in BF (2.3 [2.1; 2.6]; *p* < 0.05). No differences in the scores for crying, fussiness or sleep duration per day were observed between the three feeding groups. Overall, the numbers of infants with periods of crying of  > 2 h or fussiness for > 2 h were both low in any feeding groups (< 7% at all study visits).

AEs were reported in 65.7% of EG infants and 73.4% of CG infants, though only 4 AEs (all in EG) were considered study-product related. In BF, 76.5% had AEs (supplementary table 3, Additional file 1). The same number (*n* = 55) and percentage (38.5%) of infants in each formula group reported at least one GI disorder AE, and total occurrence of AEs of interest (lower respiratory tract infections, upper respiratory infections, otitis media) were comparable between the formula groups (supplementary table 3, Additional file 1). Any medication use was reported by 83.9% and 81.1% of the EG and CG infants, respectively, being statistically the same between EG and CG.

### Fecal microbiota

Microbiota α-diversity (Faith’s phylogenetic diversity within samples; supplementary Fig. 1A, Additional file 2) was similar among the three feeding groups at baseline and 1 month of age, but at age 2 and 3 months it was significantly lower in BF compared to CG or EG (*p* < 0.0001). At age 3 months, the between sample phylogenetic distance based on weighted UniFrac (beta-diversity) for BF vs EG samples was smaller than for BF vs CG samples (*p* = 0.045), indicating that the gut microbiota composition in EG shifted towards that of BF (supplementary Fig. 1B, Additional file 2).

**Fig. 3:**
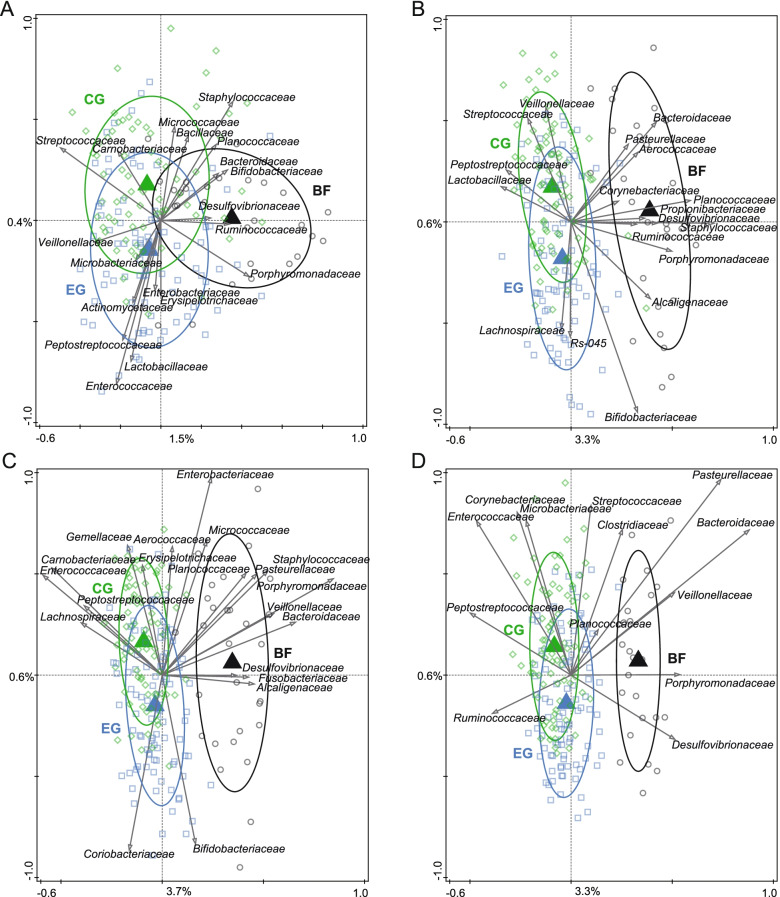
Comparison of the gut microbiota composition between the three feeding groups at baseline, 1, 2 and 3 months of age. BF, breastfed group; CG, control group; EG, experimental group. Redundancy analysis on the OTU level, assessing the effect of feeding on gut microbiota composition. OTUs were used as response data and feeding was explanatory data, the bacterial families that contributed most were plotted supplementary. The covariance attributed to confounders delivery mode and “at least 1 episode of antibiotics treatment before the 3 months visit” (only at 3 months of age) was first fitted by regression and then “partialled out” (removed) from the ordination. The (unadjusted) variation explained is indicated on the axes. **a** Baseline (≤ 0.5 months of age); variation explained by feeding was 0.8%, *p* = 0.002. **b** 1 month of age; variation explained by feeding was 2.8%, *p* = 0.002. **c** 2 months of age; variation explained by feeding was 3.3%, *p* = 0.002. **d** 3 months of age; Variation explained by feeding was 2.9%, *p* = 0.002. Sample size at each timepoint was: 71–81 in CG, 86–93 in EG and 25–29 in BF

At baseline, multivariate analysis of the microbiota composition (RDA at OTU level; corrected for delivery mode) showed no difference between the microbiota composition of EG and CG (data not shown), but a significant ordination was obtained when introducing BF (*p* = 0.002; Fig. 3A). This means BF gut microbiota at baseline was different from that of the formula-fed infants. BF was associated with e.g. *Porphyromonadaceae, Bifidobacteriaceae* and *Staphylococcaceae*, while formula-fed groups were associated with e.g. *Streptococcaceae* and *Veillonellaceae*. At 1, 2 and 3 months of age, a similar pattern was observed when including all three feeding groups in the RDA (Fig. 3B-D) with BF most different from EG and CG. However, EG and CG progressively diverged with EG moving slightly closer to BF. These microbiota changes resulted in marginally significant differences between EG and CG at 2 and 3 months of age (RDA = 0.2% with *p* = 0.050 and RDA = 0.3% with *p* = 0.052, respectively; (supplementary Fig. 2A, Additional file 2). At age 2 months, EG was associated with e.g. *Coriobacteriaceae* and *Bifidobacteriaceae*, while CG was associated with e.g. *Enterococcaceae, Enterobacteriaceae* and *Peptostreptococcaceae* (supplementary Fig. 2A, Additional file 2). At age 3 months, EG was associated with e.g. *Bifidobacteriaceae* and *Coriobacteriaceae*, while CG was associated with e.g. *Enterobacteriaceae* (among which is *Klebsiella*), *Peptostreptococcaceae* and *Lachnospiraceae* (supplementary Fig. 2B, Additional file 2).

**Fig. 4:**
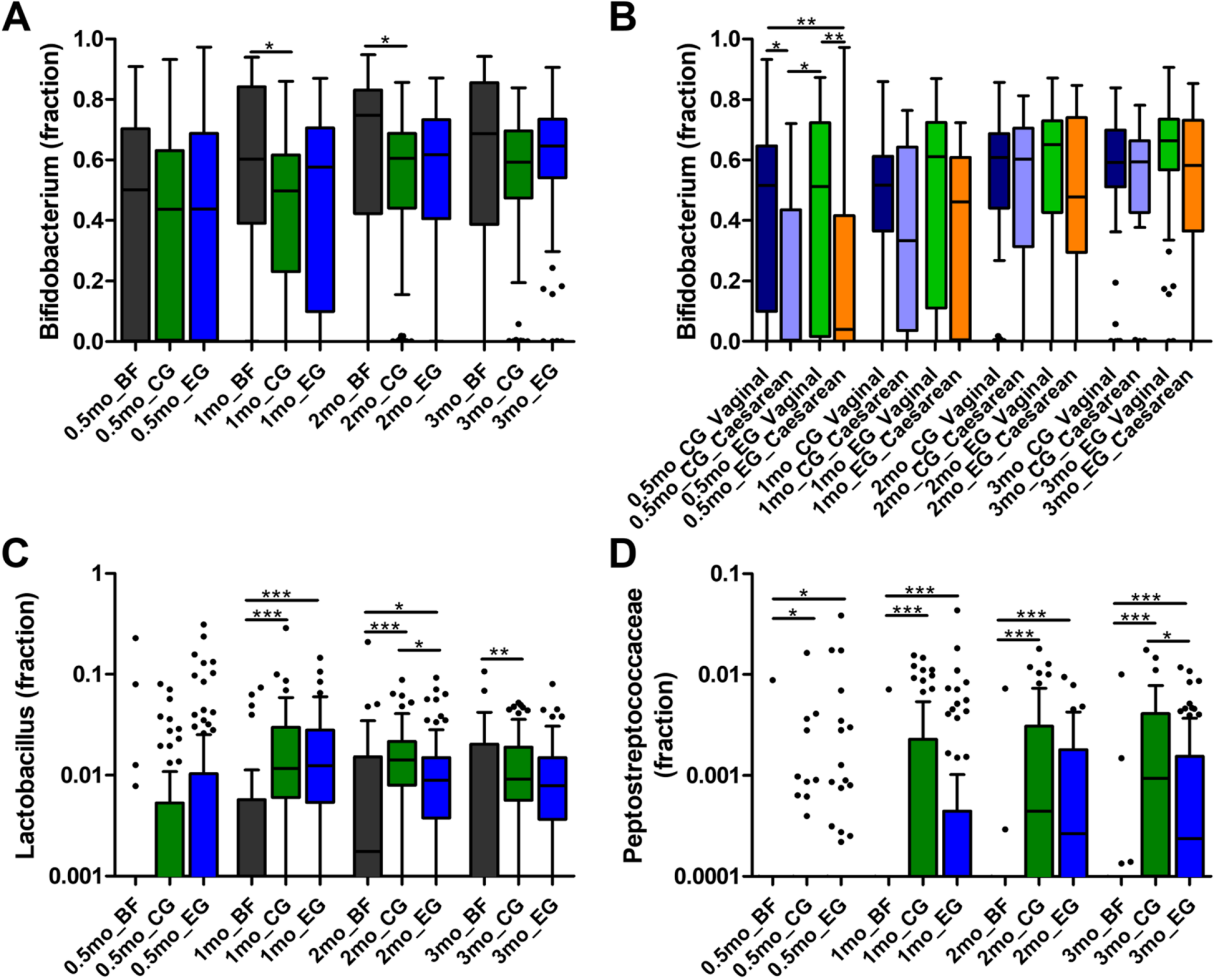
Relative abundance of **a** *Bifidobacterium* in the three feeding groups, **b** *Bifidobacterium* in the formula groups by delivery mode, **c** *Lactobacillus* and **d** *Peptostreptococcaceae* at baseline (≤ 0.5 months of age), 1, 2 and 3 months of age in the three feeding groups. BF, breastfed group; CG, control group; EG, experimental group. Groups were compared per time point by Kruskal–Wallis test, followed by pairwise comparisons with Dunn’s posthoc test. * = *p* < 0.05, ** = *p* < 0.01, *** = p < 0.001. Boxplots are displayed as Tukey whiskers. Sample size at each timepoint was: 71–81 in CG, 86–93 in EG and 25–29 in BF

*Bifidobacterium* relative abundance was significantly higher in BF compared to CG at 1 and 2 months of age. No significant differences were observed between BF and EG at any time point or between the feeding groups at age 3 months (Fig. 4A). In BF, *Lactobacillus* relative abundance was significantly lower than in CG and EG at 1 and 2 months of age, while at 3 months of age it was significantly lower in BF vs CG only. In EG compared to CG, *Lactobacillus* was comparable, except at 2 months when it was lower in EG (*p* < 0.05; Fig. 4C). *Peptostreptococcaceae* relative abundance was significantly lower in BF compared to both CG and EG at all time points. This taxon was always lower in EG compared to CG, reaching statistical significance at 3 months (Fig. 4D). Of note, *C. difficile* belongs to the *Peptostreptococcaceae* family [42].

**Fig. 5:**
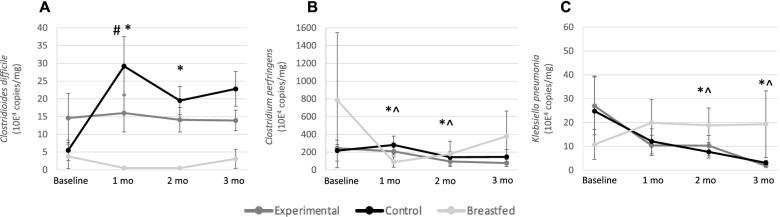
Fecal microbiota qPCR results by feeding group for **a** *Clostridioides difficile*, **b** *Clostridium perfringens*, **c** *Klebsiella pneumoniae* at baseline (≤ 14 days of age), 1, 2, and 3 months of age. Values are means of 10^4^ copies/mg fecal dry weight with SE as whiskers. Feeding groups were compared using log-transformed data in a Mixed Model Repeated Measures adjusted for baseline values, sex, mode of delivery, antibiotic use, study center, and visit. BF, breastfed group; CG, control group; EG, experimental group. # = *p* < 0.05 for EG compared to CG. * = *p* < 0.05 for CG compared to BF. ^ = *p* < 0.05 for EG compared to BF. Sample size at each timepoint was: 38–61 in CG, 47–70 in EG and 17–22 in BF for *Clostridioides difficile*; 64–74 in CG, 71–92 in EG and 23–26 in BF for *Clostridium perfringens*; 65–68 in CG, 72–84 in EG and 21–24 in BF for *Klebsiella pneumoniae*

For *C. difficile* quantified by qPCR, the mean count ± SE was significantly lower in EG than CG at 1 month (16.0 ± 5.3 × 10^4^ copies/mg vs. 29.2 ± 8.3 × 10^4^ copies/mg; *p* = 0.047) and numerically lower at 2 and 3 months (Fig. 5A). Compared with BF, counts of *C. difficile* were similar in EG at each time point, but significantly higher in CG at 1 and 2 months. For *C. perfringens,* BF showed significantly lower counts at 1 and 2 months of age, while no difference between EG and CG was observed at any time point (Fig. 5B). The abundance of *K.* *pneumoniae* was similar between EG and CG at all time points but significantly higher in BF at 2 and 3 months (Fig. 5C). Among Caesarian-born infants, counts of *K. pneumoniae* in EG were lower than in CG at 1 month (7.6 ± 5.2 × 10^4^ copies/mg vs. 37.8 ± 19.0 × 10^4^ copies/mg; *p* = 0.011). The low prevalence of EPEC, ETEC LT or ST*, Salmonella* species, C. *jejuni,* and *C. coli,* did not allow for any statistical analysis of the counts.

**Fig. 6:**
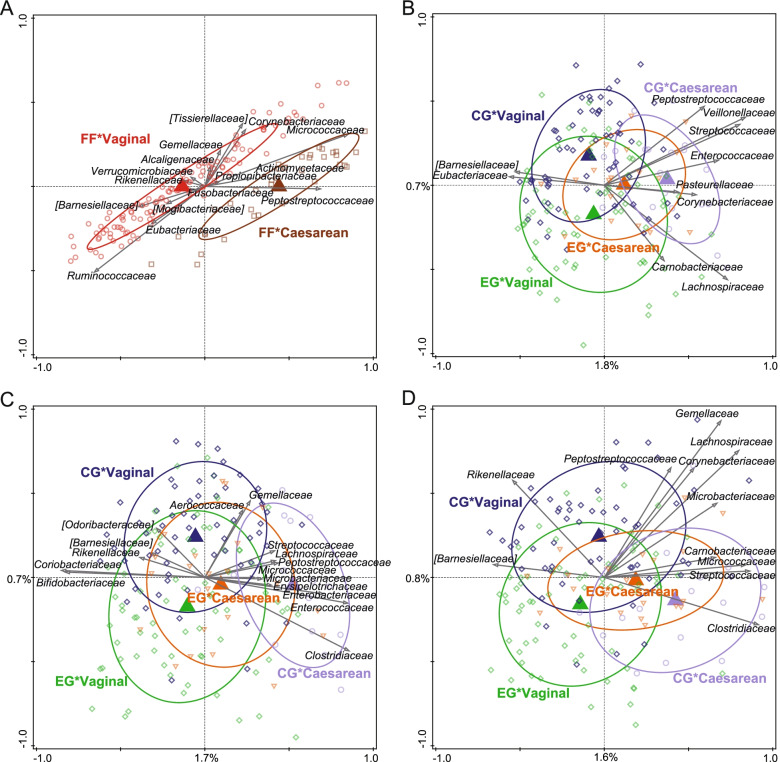
Comparison of the gut microbiota composition between the formula feeding groups at baseline, 1, 2 and 3 months of age stratified by delivery mode. CG, control group; EG, experimental group. Redundancy analysis on the OTU level at baseline, 1, 2 and 3 months of age stratified by delivery mode. OTUs were used as response data and feeding*delivery mode was explanatory data, the bacterial families that contributed most were plotted supplementary. The covariance attributed to “at least 1 episode of antibiotics treatment before the 3 months visit” (only at 3 months of age) was first fitted by regression and then “partialled out” (removed) from the ordination. The (unadjusted) variation explained is indicated on the axes. **a** Baseline (≤ 0.5 months of age). Variation explained by delivery mode was 2.7%, *p* = 0.002. **b** 1 month of age. Variation explained by feeding*delivery mode was 1.4%, *p* = 0.002. **c** 2 months of age. Variation explained by feeding*delivery mode was 1.2%, *p* = 0.002. **d** 3 months of age. Variation explained by feeding*delivery mode was 1.2%, *p* = 0.002. Sample size at each timepoint was: 71–81 in CG and 86–93 in EG

To investigate the impact of the delivery mode, we further analyzed the 16S rRNA data after stratification of the formula groups only. At baseline, before the intervention, the microbiota of the Caesarean- or vaginally born infants were significantly different (variation explained 2.7%, *p* = 0.002; Fig. 6A). At all post-baseline timepoints, the Caesarean-born infants in EG positioned slightly closer to the vaginally-born infants, compared to the Caesarean-born infants in CG (Fig. 6 B-D). Although at baseline, the relative abundance of *Bifidobacterium* was significantly lower in the Caesarean-born infants compared to vaginally-born infants, the difference rapidly faded already by 1 month of age due to an increase in the Caesarean-born infants of EG and CG (Fig. 4B).

### Fecal metabolism and markers of gut immunity and health

At baseline, fecal pH was comparable between all groups. At all post-baseline visits, BF had a lower pH compared to EG and CG (*p* < 0.001), while EG and CG were indifferent (supplementary table 4, Additional file 1). Organic acid concentrations were comparable between EG and CG. BF vs the formula groups had significantly lower concentration of acetate at 1 and 2 months, and propionate at any post-baseline visit but higher concentration of lactate at any post-baseline visit (supplementary table 4, Additional file 1). The odds of having isobutyrate or isovalerate present were significantly higher in the formula-fed groups than in BF (supplementary table 5, Additional file 1). Valerate was not detected in a sufficient number of infants for any meaningful statistical analysis.

At 2 months of age, BF (*p* = 0.043) and EF (*p* = 0.046) had slightly higher calprotectin concentration than CG. Otherwise, no differences were observed between EG and CG for any of the fecal biomarkers at any timepoint (supplementary table 6, Additional file 1). At all post baseline visits, concentrations of sIgA, myeloperoxidase, and neopterin were higher in BF than in the formula groups. At 2 months, BF had significantly higher human β-defensin-2 concentration compared to CG (supplementary table 6, Additional file 1).

## Discussion

To our knowledge, this is the first RCT comparing an experimental infant formula containing both the HMO 2’FL (1 g/L) and the probiotic *L. reuteri* DSM 17938 (1 × 10^7^ CFU/g) with a control formula containing just *L. reuteri*. Infants in EG showed comparable growth, GI tolerance and AE incidence as those in CG, indicating that the 2’FL supplemented formula containing *L. reuteri* supports age-appropriate growth, and is well-tolerated and safe. Adding 1 g/L 2’FL on top of *L. reuteri* had subtle effects on the gut microbiota, more specifically on the β-diversity moving it closer to breastfed infants, and on the abundance of opportunistic pathogens. No significant effects were observed on the GI environment or the gut maturation.

In our study, weight gain up to 4 months of age in EG was non-inferior compared to CG, in both the FAS and PP populations. Further, anthropometric z-scores were similar between formula groups and tracked closely with BF growth measures and the median of the WHO growth standards. These results are comparable to prior studies on 2’FL in combination with GOS, LNnT, or *B. lactis* Bb12 [17, 18, 21]. Our 2’FL-supplemented formula was well-tolerated. Stool consistency (indicating mushy-soft stools), stool frequency, and number of days with difficulty in passing stools did not indicate any stooling issues and no differences were seen in EG vs. CG during exclusive formula-feeding. A previous study with 2 HMOs (1 g/L 2’FL and 0.5 g/L LNnT) demonstrated significantly softer stools at 2 months of age among infants fed the test formula compared to the control [18]. Prior work has demonstrated softer stools in infants receiving a formula containing *L. reuteri* DSM 17938 [28]. In the current study, *L. reuteri* was present in both formulas which might have already impacted the stool-related outcomes in CG. This may explain why no additional improvement was observed by adding 2’FL as any incremental effect was probably not detectable with the sample size of our study. We observed similar crying, fussing, and sleeping behavioral patterns in all 3 feeding groups. The previous trial with 2 HMOs found lower rates of night time wake-ups in the HMO group compared to the control at 2 months of age and less reports of colic at 4 months of age among Cesarean-delivered infants receiving the HMO containing formula [18]. The lack of any observed difference in the current study may be due to the low incidence of prolonged crying and fussing across all feeding groups (< 7%) but may also be due to the presence of *L. reuteri* in both the EG and CG. Prior reports have shown *L. reuteri* DSM 17938 reduces crying time in breastfed infants with colic [27]. Incidence of AEs was low and comparable between EG and CG indicating that the test formula is safe. In contrast to the study investigating an infant formula supplemented with 2’FL and LNnT [18], we did not find less bronchitis and lower respiratory tract infections in the HMO group.

We did find some effects of the added 2’FL on gut microbiota. Infants in EG showed a slightly different gut microbiota profile compared to CG and we observed a marginal shift of the EG microbiota composition towards that of BF as shown by the β-diversity analysis using a metric considering the phylogenetic distances (weighted UniFrac). This means that the subtle changes of microbiota composition induced by 2’FL were better captured by high phylogenetic distances between the modulated taxa (e.g. *Bifidobacterium*, *Enterobacteriacea*, or *Peptostreptococcaceae*), reflecting their divergent functional traits in the gut ecosystem. Our RDA showed that gut microbiota in EG was associated with bifidobacteria while in CG, it was not and relative bifidobacteria abundance in EG was somewhat closer to BF compared with CG. These results are in line with in vitro work that has shown 2’FL to promote growth of *Bifidobacterium* species [44–46] and with data in breastfed infants that showed higher *Bifidobacterium* abundance in relation to 2’FL utilization [47]. On the other hand, the ability of *L. reuteri* to utilize 2’FL is negligible [48]. Compared with our study, the study with 2’FL and LNnT showed a more pronounced effect on *Bifidobacterium* abundance [19]. In our study, counts of opportunistic pathogens, namely for *C. difficile* and *K. pneumoniae,* were significantly lower in EG vs. CG at one month of age in all infants or Caesarian-born infants, respectively. These results suggest that 2’FL affected them either directly or indirectly through its effect on the gut ecology. Preclinical models and clinical observations in breastfed infants suggest that 2’FL supports the defense against pathogens, like *C. jejuni*, *E. coli*, and *P. aeruginosa*, through different mechanisms, such as prevention of pathogen adhesion or creating an unfavorable immunologic environment [49–51]. However, no specific data on preventing the binding of *C. difficile* and *K. pneumoniae* is so far available.

The microbiota results in our study should be interpreted within the context of potential effects of *L. reuteri* because, as noted above, both EG and CG received *L. reuteri*. Several prior studies have demonstrated beneficial effects of *L. reuteri* DSM 17938 on infant microbiota including increases in bifidobacteria and decreases in *Enterobacteriaceae* [25, 28, 29]. As we found a substantial *Lactobacillus* colonization in both formula-fed groups that was higher than in BF, we assume this had positive effects on the gut microbiota development and GI environment. As previously shown, *L. reuteri* can normalize the microbiota of Cesarean-born infants and promoted bifidobacteria growth in the very early days of their microbiota establishment [29]. *L. reuteri* likely plays a role of a keystone species, similar to the lactobacilli normally seeded from the vaginal microbiota. Indeed, we observed a fast recovery of bifidobacteria in both the EG and CG, and particularly in the Cesarean-born infants, making them not different from BF and indicating a possible *L. reuteri* effect. In contrast, the study with 2’FL and LNnT, found a significantly lower abundance of bifidobacteria in the control group compared to breastfed infants at 3 months of age, especially in the Cesarean-born infants [19]. This expected difference in bifidobacteria between the control and breastfed infants indicates that in absence of *L. reuteri*, there is no rapid correction of dysbiosis in Cesarean-born infants. We assume that the effect of *L. reuteri* in our CG has set the threshold higher to observe effects of 2’FL on gut microbiota and gut maturation and demonstrating strong incrementality of the 2’FL would have required a substantially higher sample size.

The higher fecal sIgA concentration observed in BF compared with the formula-fed infants can be explained by sIgA found in breast milk [52], though direct or indirect stimulatory effects of breast milk components on sIgA production in the GI mucosa likely also played a role. We also observed higher concentrations of myeloperoxidase and neopterin in BF but reference levels for these measures in infants by feeding pattern are limited. Future work is needed to evaluate concentrations of these fecal markers in infants receiving formula versus breastmilk and their potential effects on the developing immune system. We observed lower fecal pH values in the BF infants compared with those in either of the formula groups, a finding consistent with a previous infant formula trial [53]. Prior work has shown that *Bifidobacterium* abundance is inversely correlated with fecal pH which is thought to be due to consumption of HMOs and conversion to acidic end products such as acetate and lactate [47, 54]. Our findings of higher lactate and higher bifidobacteria in BF compared with EG and CG is consistent with this pathway. Our data indicates that addition of 2’FL to a formula already containing *L. reuteri* does not influence fecal pH.

Strengths of this trial include its novelty as the first RCT to compare a *L. reuteri*-supplemented control formula with the same formula additionally supplemented with 2’FL, as well as the robust sample size sufficiently powered to detect non-inferiority for the primary outcome of weight gain between the formula-fed groups. The multicenter design and enrollment of healthy term infants provided a representative sample, supporting the generalizability of the results. We also included a reference group allowing further interpretation of the data. The mothers of the breastfed reference had higher maternal education compared with the ones of the formula groups while the C-section rate was lower. These findings are likely interrelated. Mothers with a higher education more likely elect for breastfeeding and natural delivery as they are more aware of the benefits than mothers with lower education. Also, it has been shown that women who deliver by c-section are less likely to breastfeed, or delay breastfeeding initiation [55]. Finally, stool samples were collected on a monthly basis until 3 months of age, providing a good trajectory of the early life gut microbiome. The presence of *L. reuteri* in CG has limited the ability of our trial to assess the full effects of 2’FL as *L. reuteri* is known to beneficially impact infant GI symptoms and associated behaviors as well as gut microbiota. Also, we had a relatively high dropout rate independent of the feeding group, which may have limited the power of the study for the secondary outcomes, particularly the ones on microbiota, such as bifidobacteria abundance. The criteria of 80% compliant days for the PP population might appear liberal; however, we had a very strict definition of what a compliant day is (no other foods and liquids including water and tea). To avoid that too many infants would be removed from the PP population because of water and tea consumption, we did not apply a higher percentage for the compliant days.

## Conclusions

Infants fed an infant formula supplemented with 2’FL and *L. reuteri* DSM 17938 demonstrated comparable growth as infants fed a control formula supplemented with just *L. reuteri* or breastfed infants through 6 months of age. There was no difference in GI tolerance, stooling characteristics and AEs of interest between infants fed the HMO or control formula indicating that the formula with 2’FL is well-tolerated and safe. Analyses of fecal microbiota demonstrated that infants fed the 2’FL formula had a lower abundance of opportunistic pathogenic bacteria than the control during early infancy and the overall microbial pattern, including bifidobacteria abundance, in the HMO group tracked towards that of breastfed infants. This suggests that 2’FL has incremental effects on top of *L. reuteri* in infant formula and may play a role in shifting gut microbial pattern of formula-fed infant closer to that of breastfed infants.

## Supplementary Information

**Additional file 1.** 

**Additional file 2.** 

## Data Availability

All data and code used in this analysis are available from the corresponding author upon reasonable request.

## Abbreviations

2'FL: 2’-Fucosyllactose
AE: Adverse event
ANCOVA: Analysis of covariance
B. lactis: Bifidobacterium lactis
BF: Breastfed group
C. coli: Campylobacter coli
C. difficile: Clostridioides difficile
C. jejuni: Campylobacter jejuni
C. perfrigens: Clostridium perfringens
CG: Control group
CI: Confidence interval
EG: Experimental group
EPEC: Enteropathogenic Escherichia coli
ETEC: Enterotoxigenic Escherichia coli.
FAS: Full analysis set
GI: Gastrointestinal
GOS: Galactooligosaccharides
HMOs: Human milk oligosaccharides
ITT: Intention-to-treat
K. pneumonia: Klebsiella pneumonia
L. reuteri: Limosilactobacillus reuteri
LNnT: Lacto-N-neotetraose
MedDRA: Medical Dictionary for Regulatory Activities
MMRM: Mixed Model Repeated Measures
OTU: Operational taxonomic unit
PCR: Polymerase chain reaction
PP: Per protocol
qPCR: Quantitative polymerase chain reaction
RDA: Redundancy analyses
RCT: Randomized controlled
sIgA: Secretory immunoglobulin A
WHO: World Health Organization
