# Supplementary material for: Safety and efficacy of a probiotic-containing infant formula supplemented with 2’-fucosyllactose: a double-blind randomized controlled trial
Source: Nutr J. 2022 Feb 22;21:11. doi: 10.1186/s12937-022-00764-2 (PMC8862345; doi:10.1186/s12937-022-00764-2)
Supplement: Supplementary file 1 — Additional file 1. [file 12937_2022_764_MOESM1_ESM.docx]

**Supplementary File 1: Supplementary Tables**

**Article title:** Safety and efficacy of a probiotic-containing infant formula supplemented with 2’-fucosyllactose: A double-blind randomized controlled trial

**Journal:** Nutrition Journal

**Authors:** Philippe Alliet, Yvan Vandenplas, Paola Roggero, Sabine N.J. Jespers, Stefaan Peeters, Jean-Philippe Stalens, Guus A.M. Kortman, Mailis Amico, Bernard Berger, Norbert Sprenger, Colin I. Cercamondi, Giovanni Corsello

**Corresponding Author:** Colin I. Cercamondi, Nestlé Product Technology Center – Nutrition, Société des Produits Nestlé S.A., Vevey, Switzerland; ColinIvano.Cercamondi@nestle.com

**Supplementary table 1.** Stooling characteristics (stool consistency and frequency), gastrointestinal symptoms and associated behaviors by feeding group assessed throughout the study and at the post-baseline visits for the intention-to-treat population.

|  | **Experimental group (EG; n=144)** | | **Control group (CG; n=145)** | | **Breastfed group (BF; n=60)** | |
| --- | --- | --- | --- | --- | --- | --- |
|  | **LS Mean** | **95% CI** | **LS Mean** | **95% CI** | **LS Mean** | **95% CI** |
| **Stool consistency score** |  |  |  |  |  |  |
| Overall | 2.7^a^ | 2.6; 2.8 | 2.8^a^ | 2.6; 2.9 | 1.9 | 1.7; 2.1 |
| 1 month | 2.8^a^ | 2.6; 2.9 | 2.9^a^ | 2.7; 3.0 | 1.8 | 1.6; 2.0 |
| 2 months | 2.4^a^ | 2.3; 2.6 | 2.6^a^ | 2.4; 2.7 | 1.6 | 1.3; 1.8 |
| 3 months | 2.3^a^ | 2.2; 2.4 | 2.4^a^ | 2.3; 2.6 | 1.6 | 1.3; 1.8 |
| 4 months | 2.4^a^ | 2.3; 2.6 | 2.5^a^ | 2.3; 2.6 | 1.7 | 1.5; 2.0 |
| 6 months | 3.5^a^ | 3.4; 3.7 | 3.4^b^ | 3.2; 3.6 | 2.8 | 2.6; 3.1 |
| **Stool frequency** |  |  |  |  |  |  |
| Overall | 1.6^b^ | 1.5; 1.7 | 1.6^b^ | 1.5; 1.7 | 1.8 | 1.7; 2.1 |
| 1 month | 1.8^a^ | 1.7; 2.0 | 1.6^a^ | 1.5; 1.8 | 2.7 | 2.4; 3.1 |
| 2 months | 1.3^a^ | 1.2; 1.5 | 1.4^b^ | 1.2; 1.5 | 1.9 | 1.6; 2.2 |
| 3 months | 1.4 | 1.3; 1.5 | 1.4 | 1.3; 1.5 | 1.5 | 1.3; 1.8 |
| 4 months | 1.5 | 1.3; 1.6 | 1.5 | 1.4; 1.7 | 1.6 | 1.4; 1.9 |
| 6 months | 2.0 | 1.8; 2.2 | 2.1^b^ | 1.9; 2.3 | 1.7 | 1.4; 2.0 |
| **Frequency of spitting-up/vomiting** |  |  |  |  |  |  |
| Overall | 1.7 | 1.6; 1.8 | 1.6 | 1.5; 1.7 | 1.7 | 1.5; 1.9 |
| 1 month | 1.7^b^ | 1.5; 1.8 | 1.7 | 1.6; 1.9 | 2.0 | 1.7; 2.2 |
| 2 months | 1.8 | 1.6; 1.9 | 1.7 | 1.5; 1.9 | 1.7 | 1.5; 2.0 |
| 3 months | 1.8 | 1.7; 2.0 | 1.7 | 1.6; 1.9 | 1.6 | 1.4; 1.9 |
| 4 months | 1.8^c^ | 1.7; 2.0 | 1.6 | 1.5; 1.8 | 1.8 | 1.5; 2.1 |
| 6 months | 1.4^c^ | 1.3; 1.6 | 1.3^b^ | 1.1; 1.4 | 1.5 | 1.3; 1.8 |
| **Frequency of flatulence** |  |  |  |  |  |  |
| Overall | 2.7^b^ | 2.5; 2.8 | 2.7^b^ | 2.6; 2.9 | 2.3 | 2.1; 2.6 |
| 1 month | 3.0^b^ | 2.8; 3.2 | 3.1^a^ | 2.9; 3.3 | 2.4 | 2.1; 2.7 |
| 2 months | 2.9 | 2.7; 3.1 | 2.9 | 2.7; 3.1 | 2.7 | 2.4; 3.0 |
| 3 months | 2.7 | 2.5; 2.8 | 2.8 | 2.6; 3.0 | 2.5 | 2.1; 2.8 |
| 4 months | 2.5 | 2.3; 2.7 | 2.6^b^ | 2.4; 2.8 | 2.2 | 1.9; 2.5 |
| 6 months | 2.3 | 2.1; 2.5 | 2.3 | 2.1; 2.5 | 2.0 | 1.7; 2.3 |
| **Duration of crying** |  |  |  |  |  |  |
| Overall | 1.7 | 1.6; 1.9 | 1.8 | 1.7; 2.0 | 1.8 | 1.6; 2.1 |
| 1 month | 2.0 | 1.9; 2.2 | 2.2 | 2.0; 2.4 | 2.1 | 1.8; 2.4 |
| 2 months | 1.8 | 1.7; 2.0 | 1.9 | 1.8; 2.2 | 1.9 | 1.6; 2.2 |
| 3 months | 1.7 | 1.6; 1.9 | 1.8 | 1.7; 2.0 | 1.8 | 1.6; 2.1 |
| 4 months | 1.6 | 1.4; 1.7 | 1.7 | 1.5; 1.9 | 1.7 | 1.4; 2.0 |
| 6 months | 1.5 | 1.4; 1.7 | 1.5 | 1.4;1.7 | 1.8 | 1.5; 2.1 |
| **Duration of sleeping** |  |  |  |  |  |  |
| Overall | 3.0 | 2.9; 3.2 | 3.1 | 3.0; 3.2 | 3.1 | 2.9; 3.3 |
| 1 month | 3.3 | 3.2; 3.5 | 3.3 | 3.2; 3.5 | 3.4 | 3.2; 3.6 |
| 2 months | 3.1 | 3.0; 3.3 | 3.1 | 3.0; 3.3 | 3.3 | 3.1; 3.5 |
| 3 months | 3.0 | 2.8; 3.1 | 3.1 | 3.0; 3.3 | 3.2 | 2.9; 3.4 |
| 4 months | 2.9 | 2.8; 3.1 | 3.0 | 2.8; 3.2 | 2.9 | 2.7; 3.2 |
| 6 months | 2.8 | 2.7; 3.0 | 2.8 | 2.7; 3.0 | 2.7 | 2.5; 3.0 |

Superscript letters indicate statistical difference at specific post-baseline visits using a linear mixed model adjusted for baseline concentration, sex, mode of delivery, use of antibiotics, study center and visit. ^a^p<0.001 vs. BF; ^b^p<0.05 vs. BF; ^c^p<0.05 EG vs CG. A scoring approach was used to analyze the variables that were reported categorically. For frequency of spitting-up/vomiting and flatulence: 1 time/day = 1; 2-3 times = 2; 4-6 times = 3; >7 times = 4. For duration of crying: <10 min = 1; 10-30 min = 2; >30 min to 1 hour = 3; >1-2 hours = 4; >2-3 hours = 5; >3 hours = 6. For duration of sleeping: 0-8 hours = 1; 8-12 hours = 2; 12-16 hours = 3;16-20 hours = 4, 20-24 hours = 5.

**Supplementary table 2**. Odds ratios for difficulties in passing stool, fussiness duration, and severity of spitting-up/vomiting between the feeding groups assessed at all post-baseline visits for the intention-to-treat population.

|  | **Difficulty in passing stool** | | **Duration of fussiness** | | **Severity of spitting-up/vomiting** | |
| --- | --- | --- | --- | --- | --- | --- |
|  | **OR^1^** | **95%CI** | **OR^1^** | **95%CI** | **OR^1^** | **95%CI** |
| **1 month** |  |  |  |  |  |  |
| EG vs CG | 1.2 | 0.7; 2.1 | 0.8 | 0.4; 1.6 | 1.8 | 0.9; 3.6 |
| EG vs BF | 0.8 | 0.4; 1.8 | 0.9 | 0.3; 2.4 | 1.5 | 0.6; 3.8 |
| CG vs BF | 0.7 | 0.3; 1.6 | 1.1 | 0.4; 3.2 | 0.8 | 0.3; 2.1 |
| **2 months** |  |  |  |  |  |  |
| EG vs CG | 1.1 | 0.5; 2.0 | 0.5 | 0.2; 1.2 | 0.8 | 0.4; 1.7 |
| EG vs BF | 0.7 | 0.3; 1.9 | 0.6 | 0.2; 1.6 | 1.1 | 0.4; 2.9 |
| CG vs BF | 0.7 | 0.3; 1.8 | 1.0 | 0.4; 3.1 | 1.3 | 0.5; 3.8 |
| **3 months** |  |  |  |  |  |  |
| EG vs CG | 1.0 | 0.4; 2.4 | 0.8 | 0.4; 1.7 | 0.8 | 0.4; 1.7 |
| EG vs BF | 0.4 | 0.1; 1.1 | 1.0 | 0.3; 2.9 | 0.6 | 0.2; 1.9 |
| CG vs BF | 0.4 | 0.1; 1.1 | 1.2 | 0.4; 3.8 | 0.8 | 0.3; 2.4 |
| **4 months** |  |  |  |  |  |  |
| EG vs CG | 1.1 | 0.5; 2.7 | 0.5 | 0.2; 1.2 | 0.8 | 0.4; 1.8 |
| EG vs BF | 3.2 | 0.6; 16.4 | 0.8 | 0.3; 2.6 | 1.1 | 0.4; 3.3 |
| CG vs BF | 2.8 | 0.5; 14.7 | 1.6 | 0.5; 4.9 | 1.4 | 0.5; 4.0 |
| **6 months** |  |  |  |  |  |  |
| EG vs CG | 3.6^a^ | 1.4; 9.8 | 0.8 | 0.3; 2.0 | 0.9 | 0.4; 2.1 |
| EG vs BF | 0.8 | 0.3; 2.3 | 1.3 | 0.4; 4.1 | 1.4 | 0.4; 4.4 |
| CG vs BF | 0.2^a^ | 0.1; 0.8 | 1.6 | 0.5; 5.1 | 1.6 | 0.5; 5.3 |

BF, breastfed group; CG, control group; EG, experimental group; OR, odds ratio. CG, n=145; EG, n=144; BF, n=60.

^1^ OR derived from logistic regression model adjusted for baseline category, sex, mode of delivery and study center. Superscript letters indicate significantly reduced or increased OR: ^a^p<0.05. A scoring approach was used to analyze the variables that were reported categorically. For severity of spitting-up/vomiting: 1 teaspoon or less = 1; 1 tablespoon = 2; 2 tablespoons = 3; about half of the feeding = 4; more than half of the feeding = 5. For duration of fussing: <10 min = 1; 10-30 min = 2; >30 min to 1 hour = 3; >1-2 hours = 4; >2-3 hours = 5; >3 hours = 6. Scores were dichotomized (score <1.5 or score ≥1.5) as the residuals of the originally planned Mixed Model Repeated Measures were not normally distributed, even after a log-transformation of the data.

**Supplementary table 3.** Number and percentage of infants with parent-reported and physician-confirmed adverse events in the safety set of the three feeding regimens.

|  | **Experimental group (n=143)**  **n (%)** | **Control group (n=143)**  **n (%)** | **Fisher’s Exact test P-value^3^** | **Breastfed group (n=51)**  **n (%)** |
| --- | --- | --- | --- | --- |
| Any AE | 94 (65.7%)^1^ | 105 (73.4%) | N/A | 39 (76.5%) |
| Study product related AE | 4 (2.8%) | 0 (0%) | N/A | 0 (0%) |
| Any GI disorder AE^2^ | 55 (38.5%) | 55 (38.5%) | 0.14 | 16 (31.4%) |
| Diarrhea | 6 (4.2%) | 7 (4.9%) | 1 | 1 (2%) |
| Abdominal pain | 17 (11.9%) | 27 (18.9%) | 0.14 | 7 (13.7%) |
| Constipation | 11 (7.7%) | 7 (4.9%) | 0.47 | 5 (9.8%) |
| Gastroesophageal reflux disease | 12 (8.4%) | 13 (9.1%) | 1 | 2 (3.9%) |
| Regurgitation | 11 (7.7%) | 10 (7.0%) | 1 | 0 (0%) |
| Upper respiratory tract infections | 67 (46.9%) | 47 (32.9%) | 1 | 20 (39.2%) |
| Lower respiratory tract infection | 35 (24.5%) | 23 (16.1%) | 0.66 | 14 (27.5%) |
| Otitis medias | 11 (7.7%) | 7 (4.9%) | 0.21 | 7 (13.7%) |

AE, Adverse event; GI, gastrointestinal.

^1^ Number and percentage of infants with at least one AE among infants in the formula groups or breastfed group.

^2^ The five most prevalent MedDRA Preferred Term for GI disorders are listed as subcategories.

^3^ Fisher’s Exact test was used to compare formula-fed groups.

**Supplementary table 4.** Concentration of fecal pH, acetate, butyrate, lactate and propionate by feeding group assessed at four study time points for the intention-to-treat population with available fecal sample and concentrations above the detection limit.

|  | **Experimental group (EG)** | | | | **Control group (CG)** | | | | **Breastfed group (BF)** | | | |
| --- | --- | --- | --- | --- | --- | --- | --- | --- | --- | --- | --- | --- |
|  | **n** | **Mean** | **SD** | | **n** | **mean** | **SD** | | **n** | **mean** | **SD** | |
| **Fecal pH** |  |  |  | |  |  |  | |  |  |  | |
| Baseline | 101 | 6.1 | 0.68 | | 88 | 6.2 | 0.82 | | 34 | 5.7 | 0.71 | |
| 1 month | 102 | 7.1^a^ | 0.80 | | 95 | 7.2 ^a^ | 0.74 | | 41 | 6.0 | 0.76 | |
| 2 months | 100 | 7.2^a^ | 0.76 | | 79 | 7.2 ^a^ | 0.78 | | 37 | 6.0 | 0.79 | |
| 3 months | 92 | 7.3^a^ | 0.83 | | 82 | 7.3 ^a^ | 0.71 | | 27 | 6.0 | 0.88 | |
|  | **n** | **GM** | **-GSD** | **+GSD** | **n** | **GM** | **-GSD** | **+GSD** | **n** | **GM** | **-GSD** | **+GSD** |
| **Acetate** (g/kg dry feces) |  |  |  |  |  |  |  |  |  |  |  |  |
| Baseline | 99 | 8.9 | 3.8 | 20.6 | 84 | 10.3 | 5.2 | 20.5 | 35 | 9.4 | 4.3 | 20.7 |
| 1 month | 101 | 16.4^a^ | 9.4 | 28.7 | 94 | 17.1^b^ | 10.5 | 27.8 | 38 | 9.4 | 4.6 | 19.2 |
| 2 months | 97 | 19.2^b^ | 12.4 | 29.7 | 78 | 17.6^b^ | 11.1 | 28.0 | 33 | 13.5 | 7.5 | 24.4 |
| 3 months | 92 | 18.1 | 12.3 | 26.6 | 81 | 17.2 | 10.8 | 27.3 | 26 | 15.4 | 8.8 | 27.0 |
| **Butyrate** (g/kg dry feces) |  |  |  |  |  |  |  |  |  |  |  |  |
| Baseline | 31 | 0.8 | 0.2 | 3.3 | 33 | 1.1 | 0.3 | 4.5 | 7 | 1.4 | 0.6 | 3.3 |
| 1 month | 65 | 2.1 | 0.9 | 5.1 | 73 | 1.9 | 0.7 | 5.1 | 5 | 1.5 | 0.7 | 3.2 |
| 2 months | 78 | 2.1 | 0.8 | 5.3 | 65 | 2.7 | 1.2 | 6.3 | 7 | 0.5 | 0.2 | 1.2 |
| 3 months | 73 | 2.6 | 1.2 | 5.7 | 73 | 2.9 | 1.3 | 6.5 | 3 | 0.7 | 0.3 | 1.7 |
| **Lactate** (g/kg dry feces) |  |  |  |  |  |  |  |  |  |  |  |  |
| Baseline | 82 | 2.1 | 0.3 | 13.0 | 75 | 2.8 | 0.5 | 15.6 | 30 | 2.8 | 0.7 | 10.4 |
| 1 month | 72 | 0.9^b^ | 0.2 | 4.5 | 66 | 0.9^b^ | 0.2 | 5.3 | 32 | 2.7 | 0.6 | 13.5 |
| 2 months | 72 | 1.4^b^ | 0.3 | 6.2 | 55 | 1.5^b^ | 0.3 | 7.0 | 30 | 3.8 | 1.1 | 13.8 |
| 3 months | 73 | 1.1^b^ | 0.2 | 6.0 | 56 | 1.0^b^ | 0.2 | 5.6 | 25 | 5.2 | 1.7 | 16.3 |
| **Propionate** (g/kg dry feces) |  |  |  |  |  |  |  |  |  |  |  |  |
| Baseline | 73 | 2.6 | 1.2 | 5.9 | 56 | 2.1 | 0.9 | 5.3 | 15 | 0.8 | 0.4 | 1.7 |
| 1 month | 97 | 4.9^a^ | 2.5 | 9.9 | 92 | 5.0^a^ | 2.9 | 8.6 | 14 | 0.8 | 0.3 | 2.1 |
| 2 months | 94 | 5.2^a^ | 2.9 | 9.2 | 77 | 4.8^a^ | 2.9 | 8.2 | 17 | 1.7 | 0.8 | 3.7 |
| 3 months | 92 | 4.5^a^ | 2.8 | 7.3 | 79 | 5.1^a^ | 2.8 | 9.0 | 15 | 1.8 | 0.9 | 3.3 |

GM, geometric mean; GSD, geometric standard deviation. Superscript letters indicate statistical difference at specific post-baseline visits using a linear mixed model adjusted for baseline concentration, sex, mode of delivery, antibiotic use, study center and visit. ^a^p<0.001 vs BF; ^b^p<0.05 vs BF. Mixed models were applied on the log-transformation of the ratio to baseline concentration for each parameter. The low number of samples with detectable levels of butyrate at 3 months in the breastfed group did not allow any comparisons with the formula-fed infants.

**Supplementary table 5.** Odds ratios for the presence of isobutyrate and isovalerate between the feeding groups assessed at four study time points for the intention-to-treat population with available fecal sample and with concentrations above the detection limit.

|  | **Isobutyrate** | | | **Isovalerate** | | |
| --- | --- | --- | --- | --- | --- | --- |
|  | **n** | **OR^1^** | **95%CI** | **n** | **OR^1^** | **95%CI** |
| **Baseline** |  |  |  |  |  |  |
| EG vs CG | 99 (EG) | -- | -- | 99 (EG) | -- | -- |
| EG vs BF | 84 (BF) | -- | -- | 84 (BF) | -- | -- |
| CG vs BF | 35 (CG) | -- | -- | 35 (CG) | -- | -- |
| **1 month** |  |  |  |  |  |  |
| EG vs CG | 101 (EG) | 0.7 | 0.4; 1.4 | 101 (EG) | 1.0 | 0.5; 2.1 |
| EG vs BF | 94 (BF) | 4.7^a^ | 1.4; 16.2 | 94 (BF) | 29.1^b^ | 3.4; 250.4 |
| CG vs BF | 38 (CG) | 6.7^b^ | 1.9; 23.3 | 38 (CG) | 28.4^b^ | 3.3; 245.8 |
| **2 months** |  |  |  |  |  |  |
| EG vs CG | 97 (EG) | 0.6 | 0.3; 1.3 | 97 (EG) | 1.4 | 0.6; 2.9 |
| EG vs BF | 78 (BF) | 13.4^c^ | 3.1; 56.9 | 78 (BF) | 9.4^b^ | 2.2; 39.7 |
| CG vs BF | 33 (CG) | 21.7^c^ | 4.9; 94.9 | 33 (CG) | 6.9^b^ | 1.6; 30.0 |
| **3 months** |  |  |  |  |  |  |
| EG vs CG | 92 (EG) | 0.9 | 0.4; 2.0 | 92 (EG) | 1.1 | 0.5; 2.4 |
| EG vs BF | 81 (BF) | 9.0^b^ | 2.3; 35.0 | 81 (BF) | 7.8^b^ | 1.8; 33.8 |
| CG vs BF | 26 (CG) | 9.7^b^ | 2.5; 38.4 | 26 (CG) | 7.1^b^ | 1.6; 31.3 |

BF, breastfed group; CG, control group; EG, experimental group; OR, odds ratio.

^1^ OR derived from logistic regression model adjusted for baseline category, sex, mode of delivery, antibiotic use and study center. Superscript letters indicate significantly reduced or increased OR: ^a^p<0.05; ^b^p<0.01; ^c^p<0.001.

**Supplementary table 6.** Concentration of fecal biomarkers by feeding group assessed at four study time points for the intention-to-treat population with available fecal sample and concentration above the detection limit.

|  | **Experimental group (EG)** | | | | **Control group (CG)** | | | | **Breastfed group (BF)** | | | |
| --- | --- | --- | --- | --- | --- | --- | --- | --- | --- | --- | --- | --- |
|  | **n** | **GM** | **-GSD** | **+GSD** | **n** | **GM** | **-GSD** | **+GSD** | **n** | **GM** | **-GSD** | **+GSD** |
| **Secretory IgA** (mg/g dry feces) |  |  |  |  |  |  |  |  |  |  |  |  |
| Baseline | 91 | 1.8 | 0.5 | 7.0 | 81 | 1.8 | 0.4 | 7.8 | 35 | 97.3 | 47.7 | 198.4 |
| 1 month | 103 | 10.1^a^ | 3.8 | 27.2 | 96 | 12.3^a^ | 4.6 | 33.2 | 43 | 88.0 | 49.2 | 157.5 |
| 2 months | 102 | 15.7^a^ | 6.0 | 41.1 | 81 | 14.5^a^ | 5.7 | 37.0 | 37 | 71.2 | 35.8 | 141.7 |
| 3 months | 93 | 15.1^a^ | 5.4 | 42.3 | 83 | 13.3^a^ | 5.2 | 34.0 | 30 | 61.9 | 28.4 | 135.1 |
| **Myeloperoxidase** (μg/g dry feces) |  |  |  |  |  |  |  |  |  |  |  |  |
| Baseline | 102 | 2.0 | 0.6 | 7.1 | 88 | 2.4 | 0.6 | 9.0 | 35 | 3.4 | 0.8 | 14.4 |
| 1 month | 102 | 6.4^b^ | 1.6 | 25.2 | 96 | 6.4^b^ | 1.7 | 23.6 | 40 | 12.4 | 2.2 | 68.7 |
| 2 months | 101 | 8.0^b^ | 2.3 | 28.0 | 81 | 7.1^b^ | 1.8 | 28.1 | 36 | 15.8 | 4.2 | 58.9 |
| 3 months | 90 | 10.3^b^ | 2.8 | 38.2 | 82 | 7.7^b^ | 1.8 | 32.3 | 29 | 25.9 | 7.6 | 87.8 |
| **Calprotectin** (mg/g dry feces) |  |  |  |  |  |  |  |  |  |  |  |  |
| Baseline | 102 | 0.6 | 0.2 | 1.5 | 88 | 0.6 | 0.3 | 1.3 | 35 | 0.7 | 0.3 | 1.7 |
| 1 month | 102 | 1.2 | 0.6 | 2.7 | 96 | 1.2 | 0.6 | 2.3 | 43 | 0.9 | 0.4 | 2.2 |
| 2 months | 102 | 1.3^c^ | 0.6 | 2.6 | 81 | 1.0^b^ | 0.5 | 2.3 | 37 | 1.2 | 0.4 | 3.5 |
| 3 months | 93 | 1.2 | 0.5 | 2.8 | 83 | 1.0 | 0.4 | 2.4 | 30 | 1.1 | 0.4 | 3.1 |
| **Neopterin** (μg/g dry feces) |  |  |  |  |  |  |  |  |  |  |  |  |
| Baseline | 102 | 1.4 | 0.6 | 3.2 | 88 | 1.7 | 0.9 | 3.4 | 35 | 1.4 | 0.6 | 3.2 |
| 1 month | 103 | 1.6^b^ | 0.9 | 3.0 | 94 | 1.7^b^ | 0.9 | 3.1 | 43 | 2.0 | 0.9 | 4.3 |
| 2 months | 102 | 2.1^b^ | 1.2 | 3.7 | 81 | 2.3^b^ | 1.3 | 4.1 | 36 | 2.7 | 1.3 | 5.4 |
| 3 months | 93 | 2.6^b^ | 1.5 | 4.5 | 83 | 2.3^a^ | 1.4 | 3.8 | 30 | 3.4 | 1.8 | 6.3 |
| **Human β-defensin-2** (μg/g dry feces) |  |  |  |  |  |  |  |  |  |  |  |  |
| Baseline | 102 | 0.5 | 0.2 | 1.1 | 88 | 0.5 | 0.2 | 1.2 | 35 | 0.4 | 0.2 | 0.9 |
| 1 month | 103 | 0.7 | 0.3 | 1.9 | 96 | 0.6 | 0.2 | 1.6 | 43 | 0.7 | 0.3 | 1.6 |
| 2 months | 102 | 0.7 | 0.3 | 1.7 | 81 | 0.6^b^ | 0.3 | 1.3 | 37 | 0.9 | 0.4 | 2.1 |
| 3 months | 93 | 0.7 | 0.3 | 1.6 | 83 | 0.7 | 0.3 | 1.5 | 30 | 0.9 | 0.3 | 2.4 |

GM, geometric mean; GSD, geometric standard deviation; IgA, Immunoglobulin A.

Superscript letters indicate statistical difference at specific post-baseline visits using a linear mixed model adjusted for baseline concentration, sex, mode of delivery, study center and visit. ^a^p<0.001 vs BF; ^b^p<0.05 vs. BF; ^c^p<0.05 EG vs CG.

Mixed models were applied on the log-transformation of the ratio to baseline concentration for each parameter.
