# Supplementary material for: Safety and efficacy of a probiotic-containing infant formula supplemented with 2’-fucosyllactose: a double-blind randomized controlled trial
Source: Nutr J. 2022 Feb 22;21:11. doi: 10.1186/s12937-022-00764-2 (PMC8862345; doi:10.1186/s12937-022-00764-2)
Supplement: Supplementary file 2 — Additional file 2. [file 12937_2022_764_MOESM2_ESM.pptx]

## Slide 1
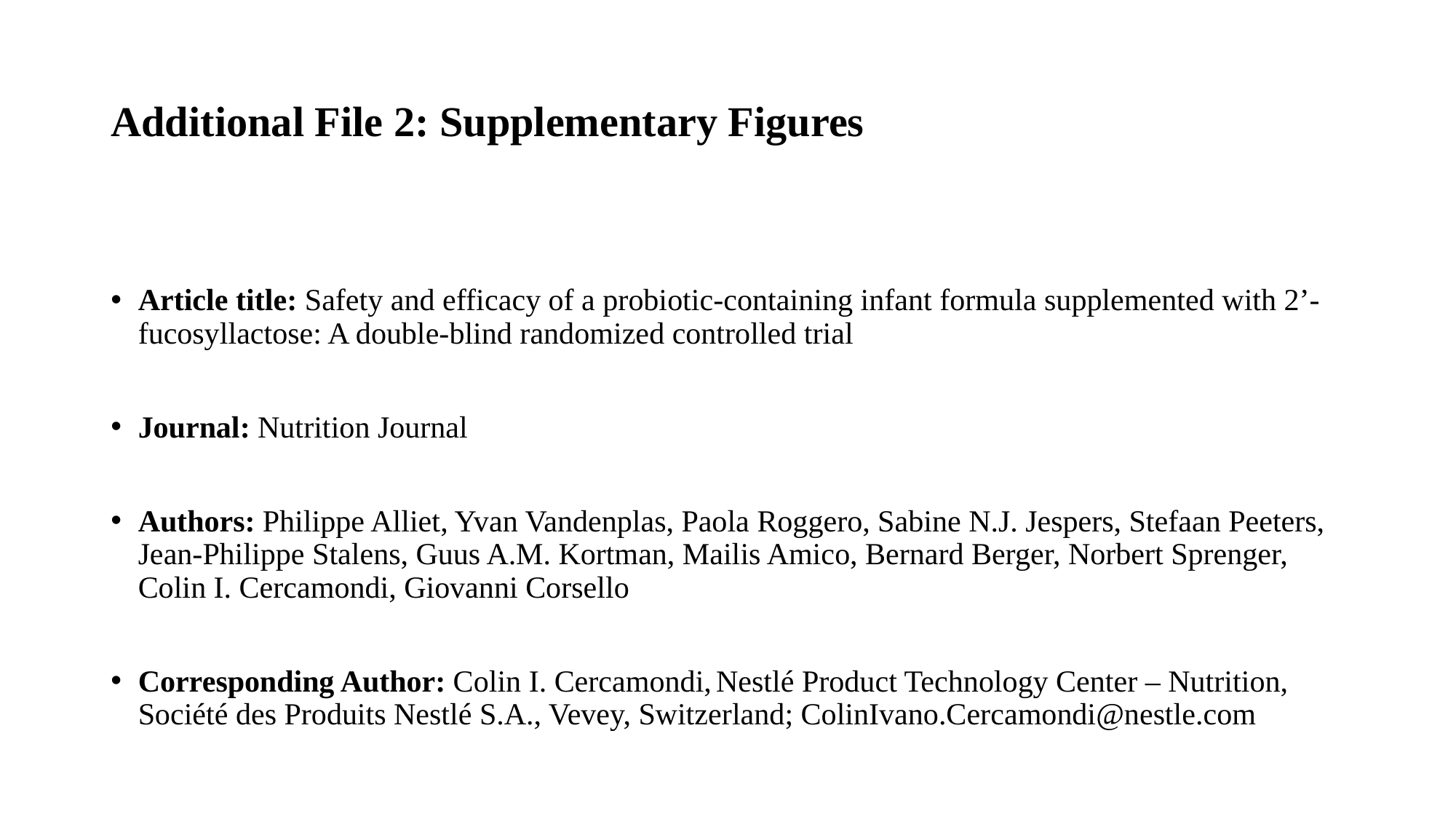

# Additional File 2: Supplementary Figures
Article title: Safety and efficacy of a probiotic-containing infant formula supplemented with 2’-fucosyllactose: A double-blind randomized controlled trial
Journal: Nutrition Journal
Authors: Philippe Alliet, Yvan Vandenplas, Paola Roggero, Sabine N.J. Jespers, Stefaan Peeters, Jean-Philippe Stalens, Guus A.M. Kortman, Mailis Amico, Bernard Berger, Norbert Sprenger, Colin I. Cercamondi, Giovanni Corsello
Corresponding Author: Colin I. Cercamondi, Nestlé Product Technology Center – Nutrition, Société des Produits Nestlé S.A., Vevey, Switzerland; ColinIvano.Cercamondi@nestle.com

## Slide 2
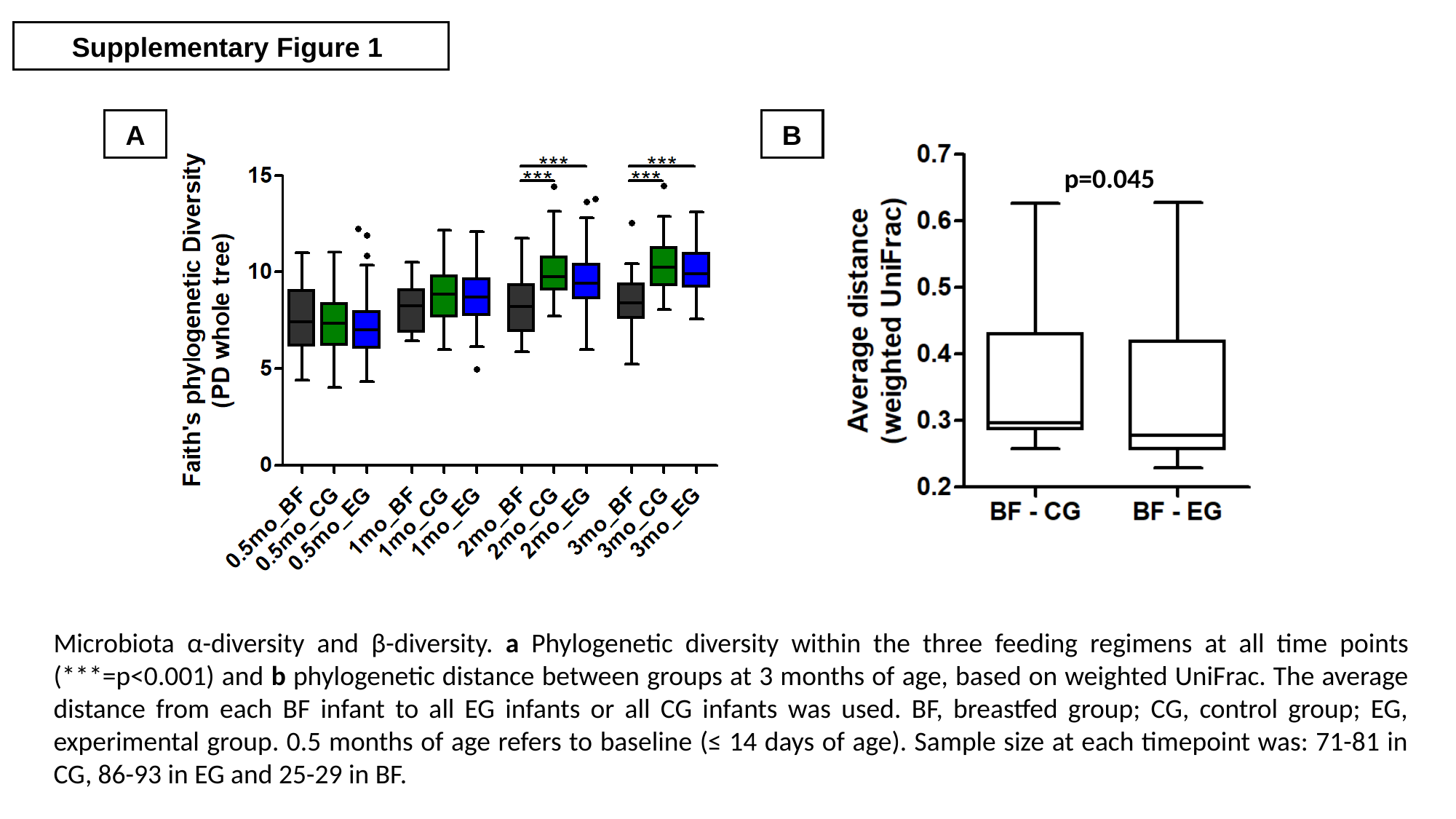

Supplementary Figure 1
A
B
p=0.045
Microbiota α-diversity and β-diversity. a Phylogenetic diversity within the three feeding regimens at all time points (***=p<0.001) and b phylogenetic distance between groups at 3 months of age, based on weighted UniFrac. The average distance from each BF infant to all EG infants or all CG infants was used. BF, breastfed group; CG, control group; EG, experimental group. 0.5 months of age refers to baseline (≤ 14 days of age). Sample size at each timepoint was: 71-81 in CG, 86-93 in EG and 25-29 in BF.

## Slide 3
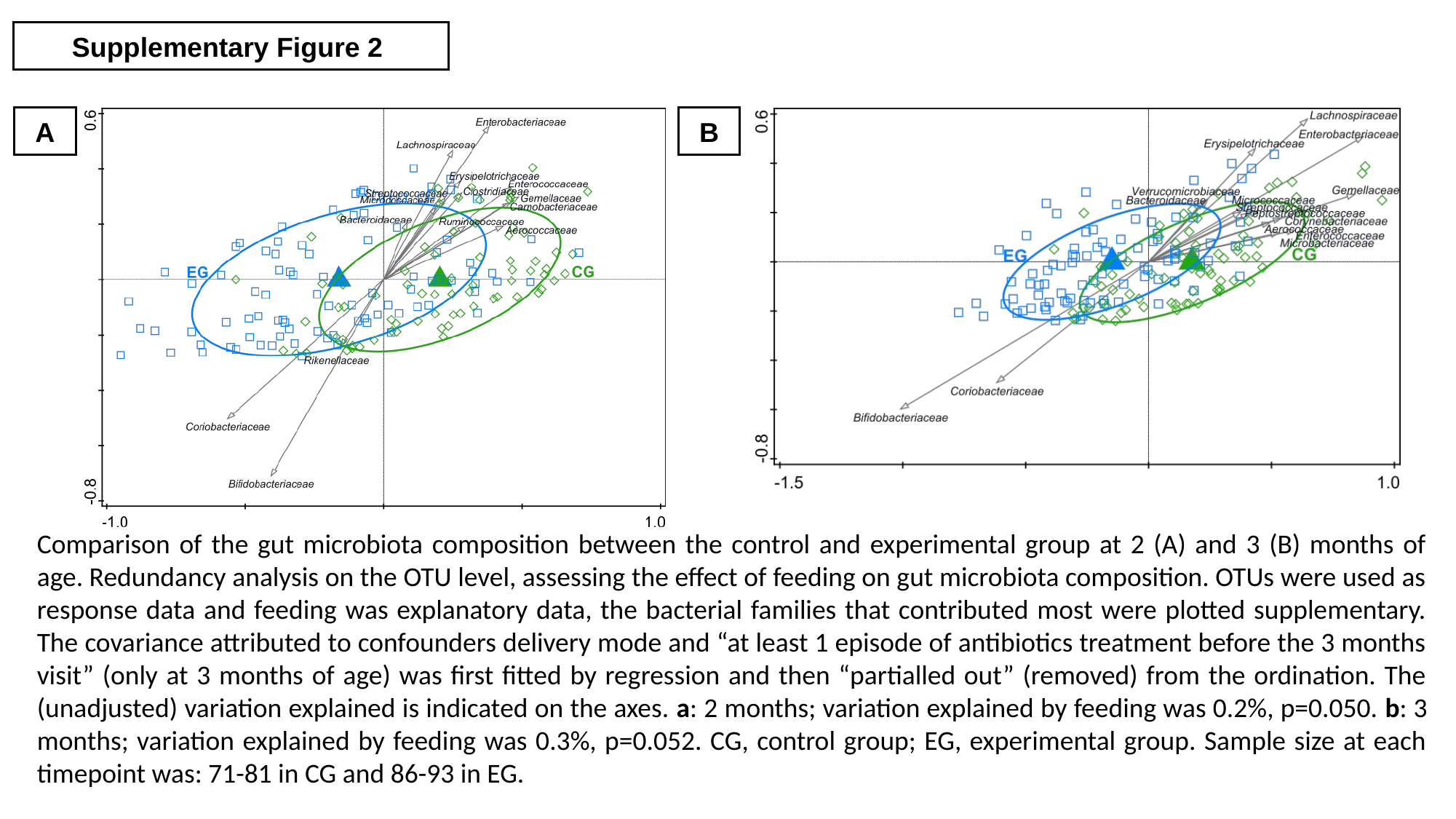

Supplementary Figure 2
A
B
Comparison of the gut microbiota composition between the control and experimental group at 2 (A) and 3 (B) months of age. Redundancy analysis on the OTU level, assessing the effect of feeding on gut microbiota composition. OTUs were used as response data and feeding was explanatory data, the bacterial families that contributed most were plotted supplementary. The covariance attributed to confounders delivery mode and “at least 1 episode of antibiotics treatment before the 3 months visit” (only at 3 months of age) was first fitted by regression and then “partialled out” (removed) from the ordination. The (unadjusted) variation explained is indicated on the axes. a: 2 months; variation explained by feeding was 0.2%, p=0.050. b: 3 months; variation explained by feeding was 0.3%, p=0.052. CG, control group; EG, experimental group. Sample size at each timepoint was: 71-81 in CG and 86-93 in EG.
